# “Executive functions” cannot be distinguished from general intelligence: two variations on a single theme within a symphony of latent variance

**DOI:** 10.3389/fnbeh.2014.00369

**Published:** 2014-10-24

**Authors:** Donald R. Royall, Raymond F. Palmer

**Affiliations:** ^1^Department of Psychiatry, The University of Texas Health Science Center, San AntonioSan Antonio, TX, USA; ^2^Department of Medicine, The University of Texas Health Science Center, San AntonioSan Antonio, TX, USA; ^3^Department of Family and Community Medicine, The University of Texas Health Science Center, San AntonioSan Antonio, TX, USA; ^4^The South Texas Veterans' Health System Audie L. Murphy Division, Geriatric Research Education and Clinical CenterSan Antonio, TX, USA

**Keywords:** aging, cognition, dementia, executive function, functional status, g, intelligence

## Abstract

The empirical foundation of executive control function (ECF) remains controversial. We have employed structural equation models (SEM) to explicitly distinguish domain-specific variance in executive function (EF) performance from memory (MEM) and shared cognitive performance variance, i.e., Spearman's “g.” EF does not survive adjustment for both MEM and g in a well fitting model of data obtained from non-demented older persons (*N* = 193). Instead, the variance in putative EF measures is attributable only to g, and related to functional status only through a fraction of that construct (i.e., “d”). d is a homolog of the latent variable δ, which we have previously associated specifically with the Default Mode Network (DMN). These findings undermine the validity of EF and its putative association with the frontal lobe. ECF may have no existence independent of general intelligence, and no functionally salient association with the frontal lobe outside of that structure's contribution to the DMN.

## Introduction

Executive Control Function (ECF) is widely thought to be vital to human autonomy, and a major determinant of problem behavior and disability in neuropsychiatric disorders (Royall et al., 2002a). Nevertheless, we lack a “gold standard” ECF measure, and the construct as a whole seems to lack a coherent empirical foundation.

“Executive functions” (EF) broadly encompass cognitive skills that are responsible for the planning, initiation, sequencing, and monitoring of complex goal-directed behavior. This may explain the relatively robust associations between EF measures and Instrumental Activities of Daily Living (IADL) (Royall et al., 2007).

However, the relationship between EF and functional status is more complex. Individual EF measures empirically load on more than one “executive” factor (Miyake et al., 2000; Royall et al., 2003; Androver-Roig et al., 2012; Testa et al., 2012). Neither the EF factor nor their indicators are necessarily associated with IADL. Executive measures are therefore commonly “validated” against structural or functional frontal lobe pathology. However, these associations are statistically weak to moderate, and qualitatively non-specific. Many executive tasks and measures can be associated with non-frontal structures and lesions (Collette and Van der Linden, 2002; Alvarez and Emory, 2006).

Recently, my colleagues and I have examined the “cognitive correlates of functional status” as a latent variable (i.e., “δ” for “dementia”) in a Structural Equation Model (SEM) framework (Royall and Palmer, 2012, 2013, 2014; Royall et al., 2012a,b, 2013). δ and its homologs are strongly associated with IADL, more strongly so than are any of their indicators, including EF measures.

δ's design explicitly parses a battery's shared variance (i.e., Spearman's g) into orthogonal fractions (g′ and δ) of which only δ is related to functional status (i.e., δ's “target indicator”) (Royall and Palmer, 2012). δ “homologs” can be constructed from any battery that contains both cognitive measures and one or more measures of IADL.

By definition, dementia requires disabling cognitive impairment. Therefore, only δ's variance is both necessary and sufficient to dementia case finding. Thus, δ scores can be interpreted as a dementia phenotype. δ homologs have achieved Areas Under the Receiver Operating Curve (AUC /ROC) of 0.92–0.99 for the discrimination of well-characterized Alzheimer's Disease (AD) cases vs. controls in four datasets to date, although each δ homolog accounts for a minority of the variance in observed cognitive performance. The latent variable g′ (δ's residual in Spearman's g) and measurement “error” (including domain specific variance) account for the majority of cognitive variance, yet g′ has an AUC of only 0.52–0.66 (Royall et al., 2012a,b, 2013; Royall and Palmer, 2013, 2014). δ has been independently validated by a second group using the National Alzheimer's Coordinating Center's (NACC) Unified Dataset (UDS) (Gavett et al., 2014). In that dataset (*N* ≈ 26,000), δ had an AUC of 0.96 for the discrimination between demented and non-demented participants, vs. g′ s 0.52. It is important to note that the NACC dataset is not limited to AD, but includes cases with a variety of dementing illnesses. This supports δ's association with dementia in the abstract, regardless of its etiology.

δ and its homologs are derived from Spearman's general intelligence factor, “g” (Spearman, 1904), i.e., a latent variable representing the shared variance in the dominant factor extracted from any cognitive battery. The latent variable g, in turn, has been associated with frontal lobe lesions (Duncan and Owen, 2000; Duncan et al., 2000) executive measures (Duncan et al., 1997), and frontal lobe imaging (Choi et al., 2008; Gläscher et al., 2010). Since “g” can also be associated with functional outcomes (Gottfredson, 1997), we decided to explore whether an EF specific factor can be distinguished from other domain-specific variance (i.e., memory) and/or g-δ. If not, then EF may merely represent g or δ's influence on cognitive task performance, and δ may represent the emergent “ECF” responsible for uniquely human “executive” capacities.

## Methods

### Air force villages' freedom house study

We have studied 547 well elderly retirees as part of the Air Force Villages' (AFV) Freedom House Study (FHS). The AFV is a 1500-bed Comprehensive Care Retirement Community in San Antonio, TX that is open to Air Force officers and their dependents. At baseline, the FHS subjects represented a random sample of AFV residents over the age of 70 years living at non-institutionalized levels of care. Informed consent was obtained prior to their evaluations.

A subset of FHS participants (*n* = 193) were administered a formal neuropsychological test battery that included standardized tests of memory, language, and ECF. This subgroup was slightly older at baseline than the larger FHS cohort (mean age of 79.0 years vs. 77.7 years, respectively), but did not differ significantly with regard to gender, education, baseline level of care, or Mini-Mental State Examination (MMSE) scores (Folstein et al., 1975). Select demographic and clinical features are presented in Table 1.

**Table 1:** **Subject characteristics**.

**Variable**	***N* = 193**
AGE (years)	77.9 (4.9)
Education (years)	15.1 (2.4)
IADL total (MAX = 14.0)	13.3 (1.7)
ADL total (MAX = 14.0)	13.5 (1.4)
MD visits previous 6 months	3.8 (4.1)
VISION (2 = “good”; 3 = “fair”)	2.2 (0.8)
% Female	58.3
% Living alone	27.8
% Reporting	
Glaucoma	18.3
Arthritis	61.2
HTN	41.9
CAD	22.5
CVA	6.3
AODM	5.6
% Using prostheses	12.0

*ADL's, activities of daily living; AODM, adult onset diabetes mellitus; CAD, coronary artery disease; CVA, cerebrovascular disease, IADL's, Instrumental Activities of Daily Living; HTN, hypertension; MAX, maximum; MD, physician*.

### Cognitive battery

#### Memory measures

The California Verbal Learning Task (CVLT) (Delis et al., 1987) assesses learning and memory processes. Patients are asked to learn and recall two 16 item shopping lists. Each list is comprised of four words from four semantic categories. Learning takes place over five trial presentations. We modeled the summed number of correct words recalled across learning trials 1–5.

The Mattis Dementia Rating Scale: memory subscale (DRS:MEM) (Mattis, 1988) provides a brief assessment of verbal and nonverbal short-term memory. The memory subtest consists of sentence (five word) recall, design and word recognition, and orientation items.

#### “Executive” measures

CLOX: An Executive Clock Drawing Task (Royall et al., 1998b) is a brief ECF measure based on a clock-drawing task (CDT). It is divided into two parts. CLOX1 is an unprompted task that is sensitive to executive control. CLOX2 is a copied version that is less dependent on executive skills. Each CLOX subtest is scored on a 15-point scale. Lower CLOX scores are impaired.

The Executive Interview (EXIT25) (Royall et al., 1992) provides a standardized clinical EF assessment. It contains 25 items designed to elicit signs of frontal system pathology (e.g., imitation, intrusions, disinhibition, environmental dependency, perseveration, and frontal release). EXIT25 scores range from 0 to 50. High scores indicate impairment.

The Controlled Oral Word Association (COWA) (Benton and Hamsher, 1989) is a test of oral word production (verbal fluency). The patient is asked to say as many words as they can, beginning with a certain letter of the alphabet.

The WAIS-R Digit Symbol Coding (DSS) (Wechsler, 1991) is a test of psychomotor speed and attentional control the subject is asked to copy as quickly as possible, nonsense symbols corresponding to specific numbers presented in a “key” at the top of the page.

The Trail Making Test, Parts A and B (Reitan, 1958) provide a measure of conceptualization, psychomotor speed, and attention. Trails B requires the subject to connect consecutively numbered and lettered circles, alternating between the two sequences.

The abbreviated Wisconsin Card Test (Haaland et al., 1987) is an adaptation of the original two deck (128 cards) Wisconsin Card Sorting Test (WCST) (Heaton et al., 1993). The Abbreviated WCST utilizes one deck of 64 cards. The number of “categories correct” (WCAT) was used as an outcome measure.

Although the above are all widely considered to be validated “executive” measures, they empirically load on at least three factors (Royall et al., 2003).

### Functional status

Disability and comorbid medical conditions were assessed using the Older Adults Resources Scale (OARS) (Fillenbaum, 1978). The OARS is a structured clinical interview that provides self-reported information on activities of daily living (ADL), IADL, physical and mental health history, healthcare utilization, and current medications.

### Statistical approach

This analysis was performed using Analysis of Moment Structures (AMOS) software (Arbuckle, 2006). All analyses were conducted in an SEM framework.

#### Analysis sequence

First we examined the associations between individual cognitive performance measures and IADL in a multivariate regression model, adjusted for age, education, and gender. The covariates were entered first, and their effect on IADL established. Then the entire set of cognitive performance measures was added as predictors. IADL was used as the dependent variable. Model fit was examined.

Next, we reorganized the observed variables as a confirmatory bifactor measurement model, testing our apriori assumptions about which measures can be associated with domain specific “memory” and “executive” factors (i.e., “MEM” and “ECF,” respectively). All indicators were adjusted for age, education, and gender. The relative correlations between both latent constructs and IADL were determined. Model fit was again examined.

Next, we introduced a third latent construct representing Spearman's general intelligence factor “g.” The entire battery of psychometric measures was used as g's indicators. We examined the effect of g's introduction the latent domain specific factors and their indicator weights. As before, all indicators were also adjusted for age, education, and gender. The relative correlations between g, the domain specific latent constructs and IADL were determined. Model fit was again examined.

Next, we reorganized g into IADL-related and independent fractions (i.e., “d” and “g,” respectively), as previously described (e.g., Royall and Palmer, 2013). By definition, g′ had no association with IADL. The relative correlations between d, the domain specific latent constructs and IADL were determined. Model fit was again examined.

Next, we searched for additional measure specific associations between individual cognitive measures and IADL, independent of the latent constructs. Finally, we systematically explored the possibility of significant intercorrelations amongst the indicator variables' residuals, which might suggest the existence of additional latent constructs other than g′, d, MEM, and EF. Only intercorrelations between two indicators' residuals that were statistically significant, improved model fit and did not result in negative variance or other model misspecifications, were retained.

#### Missing data

These models were all constructed in an SEM framework, using raw data. Modern Missing Data Methods were automatically applied by the AMOS software. AMOS uses Full information Maximum Likelihood (FIML) methods to address missing data. FIML uses the entire observed data matrix to estimate parameters with missing data. In contrast to list wise or pair wise deletion, FIML yields unbiased parameter estimates, preserves the overall power of the analysis, and is arguably superior to alternative methods, e.g., multiple imputation (Schafer and Graham, 2002; Graham, 2009).

#### Fit indices

Model fit was assessed using four common test statistics: chi-square, the ratio of the chi-square to the degrees of freedom in the model (CMIN /DF), the comparative fit index (CFI), and the root mean square error of approximation (RMSEA). Where two nested models were compared, the Browne–Cudek Criterion (BCC) was added (Browne and Cudeck, 1989).

A non-significant chi-square signifies that the data are consistent with the model (Bollen and Long, 1993). However, in large samples, this metric is limited by its tendency to achieve statistical significance when all other fit indices (which are not sensitive to sample size) show that the model fits the data very well. A CMIN/DF ratio <5.0 suggests an adequate fit to the data (Wheaton et al., 1977). The CFI statistic compares the specified model with a null model (Bentler, 1990). CFI values range from 0 to 1.0. Values below 0.95 suggest model misspecification. Values approaching 1.0 indicate adequate to excellent fit. An RMSEA of 0.05 or less indicates a close fit to the data, with models below 0.05 considered “good” fit, and up to 0.08 as “acceptable” (Browne and Cudeck, 1993). A lower BCC statistic indicates better fit (Browne and Cudeck, 1989). All fit statistics should be simultaneously considered when assessing the adequacy of the models to the data.

## Results

**Figure 1:**
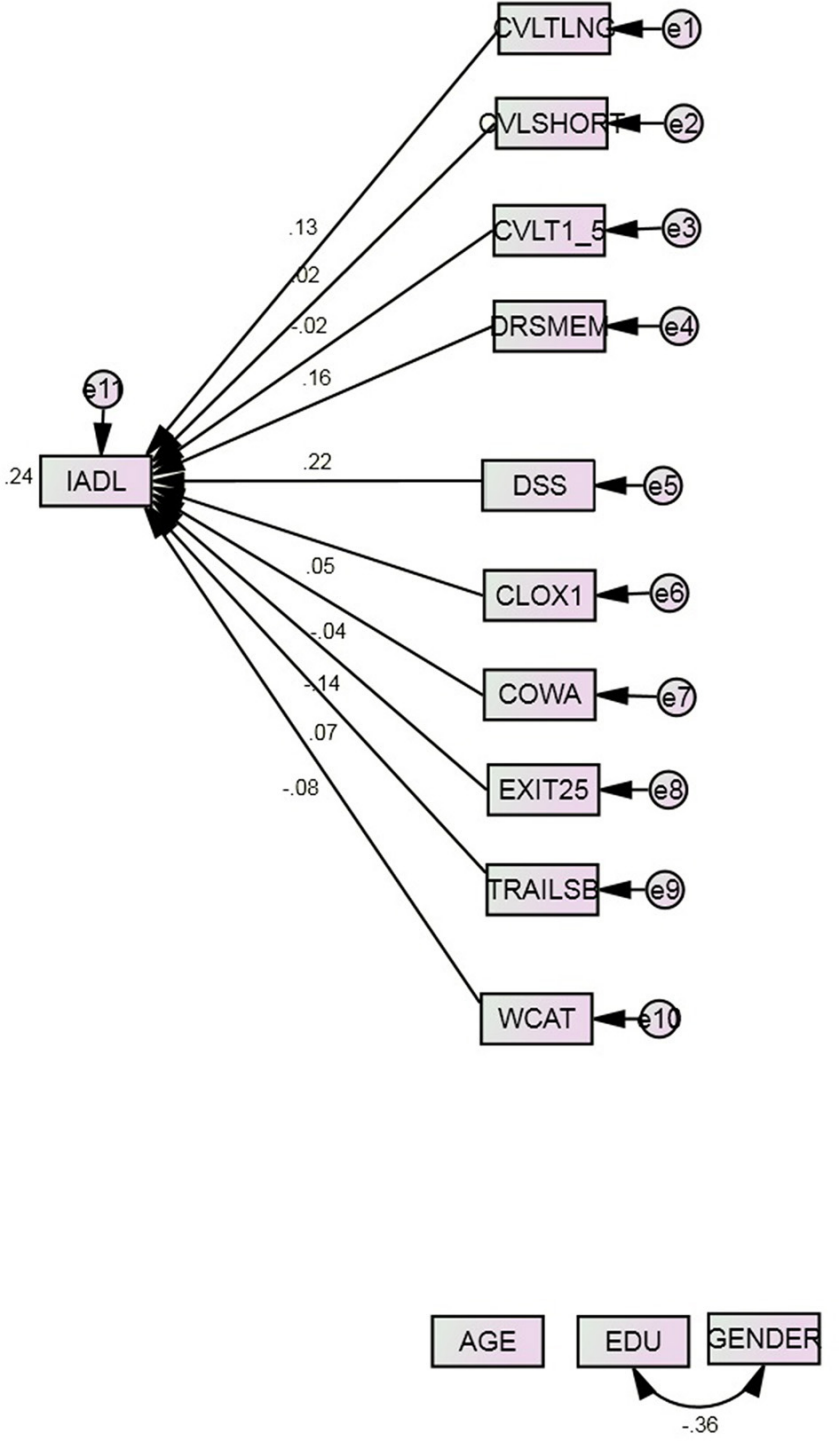
Model 1^*^. COWA, Controlled Oral Word Association Test; CLOX1, clock drawing to command; CVLT, California Verbal Learning Test; 1–5, Summed learning trials 1–5, SHORT, immediate recall, LNG, delayed recall; DRS: MEM, Mattis Dementia Rating Scale Memory subscale; DSS, WAIS-R Digit Symbol Substitution; EDU, Education; EXIT25, Executive Interview; IADL, Instrumental Activities of Daily Living; TrailsB, Trail-making Test Part B; WCAT, Wisconsin Card Sorting Test categories achieved. ^*^All observed indicators are adjusted for age, education, and gender (paths not shown).

Sample demographics are presented in Table 1. Clinical assessment means are presented in Table 2. Model 1's fit was poor (Table 3). Together, the cognitive performance measures and covariates explained 24.1% of variance in IADL. Age, gender, DSS (*r* = 0.224, *p* = 0.001) DRS:MEM (*r* = 0.158, *p* = 0.02) and EXIT25 (partial *r* = −0.145, *p* < 0.001), contributed significantly to IADL, similar to previous analyses in this cohort (Royall et al., 2000, 2004, 2005a,b) (Figure 1).

**Table 2:** **Raw cognitive performance means**.

**Variable (*N* = 193)**	**Mean (*SD*)**
**MEMORY TESTS**	
CVLT: 1–5	32.8 (14.1)
CVLT: Long	16.0 (3.7)
CVLT: Short	15.0(3.8)
DRS: MEM	21.5 (4.3)
**ECF TESTS**	
CLOX1	10.1 (3.3)
COWA	32.0 (12.6)
DSS	33.2 (11.6)
EXIT25	14.6 (5.5)
Trails B (s)	132.8 (80.8)
WCAT	2.0 (2.0)

*COWA, Controlled Oral Word Association Test; CLOX1, clock drawing to command; CVLT, California Verbal Learning Test, List A delayed recall; DRS: MEM, Mattis Dementia Rating Scale Memory subscale; DSS, WAIS-R Digit Symbol Substitution; EXIT25, Executive Interview; Trails B, Trail-making Test Part B; WCAT, Wisconsin Card Sorting Test categories achieved*.

**Table 3:** **Model fit**.

**Model**	**χ^2^ (*df*), *p***	**CFI**	**RMSEA**	**BCC**
1	881.48 (47), *p* < 0.001	0.323	0.169	1029.03
2	142.15 (45), *p* < 0.001	0.921	0.059	293.80
3a	73.06 (39), *p* = 0.001	0.972	0.037	237.00
3b	53.99 (41), *p* = 0.08	0.989	0.023	213.83
4	36.12 (31), *p* = 0.24	0.996	0.016	216.46

*BCC, Browne–Cudek Criterion; CFI, Comparative Fit Index; RMSEA, Root Mean Square Error of Approximation*.

**Figure 2:**
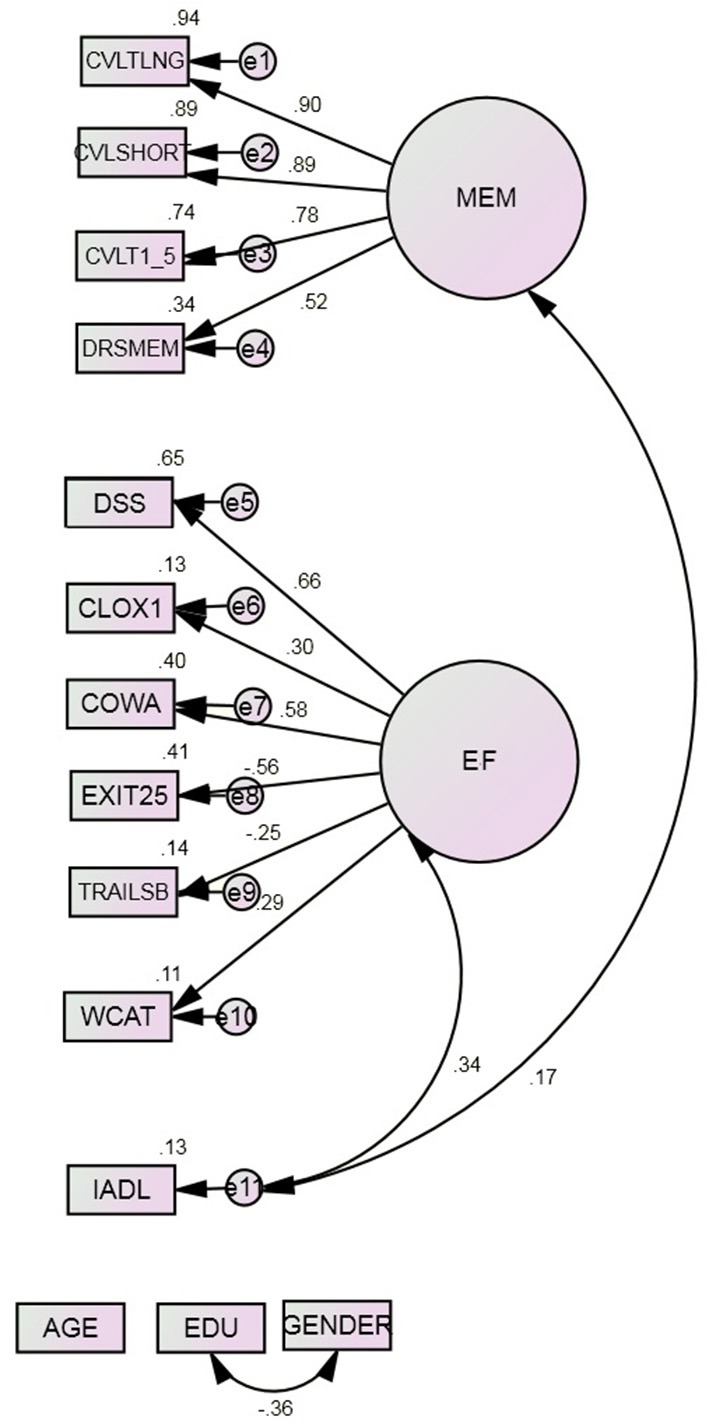
Model 2^*^. COWA, Controlled Oral Word Association Test; CLOX1, clock drawing to command; CVLT, California Verbal Learning Test; 1–5, Summed learning trials 1–5, SHORT, immediate recall, LNG, delayed recall; DRS: MEM, Mattis Dementia Rating Scale Memory subscale; DSS, WAIS-R Digit Symbol Substitution; EDU, Education; EXIT25, Executive Interview; IADL, Instrumental Activities of Daily Living; TrailsB, Trail-making Test Part B; WCAT, Wisconsin Card Sorting Test categories achieved. ^*^All observed indicators are adjusted for age, education, and gender (paths not shown).

Model 2 posits two domain specific factors, MEM and EF (Figure 2). The fit of this model is significantly improved relative to Model 1 (Table 3). CVLT:Short, CVLT:Long, CVLT 1–5, and DRS MEM all load significantly on MEM (all *p* < 0.001). The strengths of their loadings ranged from *r* = 0.52 (DRS:MEM) to *r* = 0.90 (CVLT:Long). CLOX1, DSS, EXIT25, Trails B, and WCAT all load significantly on EF (all *p* = 0.002). The strengths of their loadings ranged from *r* = −0.25 (Trails B) to 0.66 (DSS). MEM and EF were uncorrelated. As expected, EF was significantly associated with IADL (*r* = 0.34, *p* < 0.001). MEM was weakly but significantly correlated with IADL independent of EF (*r* = 0.17, *p* = 0.02).

Model 3 posited the addition of a third factor, Spearman's g. Our first attempt at a three factor model failed (due to unsuccessful minimization and negative variance). Minimization could be achieved by correlating EF and MEM but (1) the correlation between MEM and EF was not significant (*r* = −0.04, *p* = 0.923), (2) negative variance persisted on COWA's residual, (3) EF lost its association with IADL (*r* = −0.05, *p* = 0.924), (4) EF had no significant indicators (all *p* > 0.92).

Two alternative two factor models were then tested. Model 3a omitted the factor MEM (Table 3). Model 3b omitted the factor EF. In each case, these models containing g fit the data better than Models 1 or 2. In each case, the latent variable g had a stronger correlation with IADL than did the second domain specific factor. In Model 3a, g fully mediated EF's previously significant association with IADL in Model 2. However, Model 3b fit the data significantly better than did Model 3a. On the basis of these findings, the latent factor EF was deleted from subsequent models.

**Figure 3:**
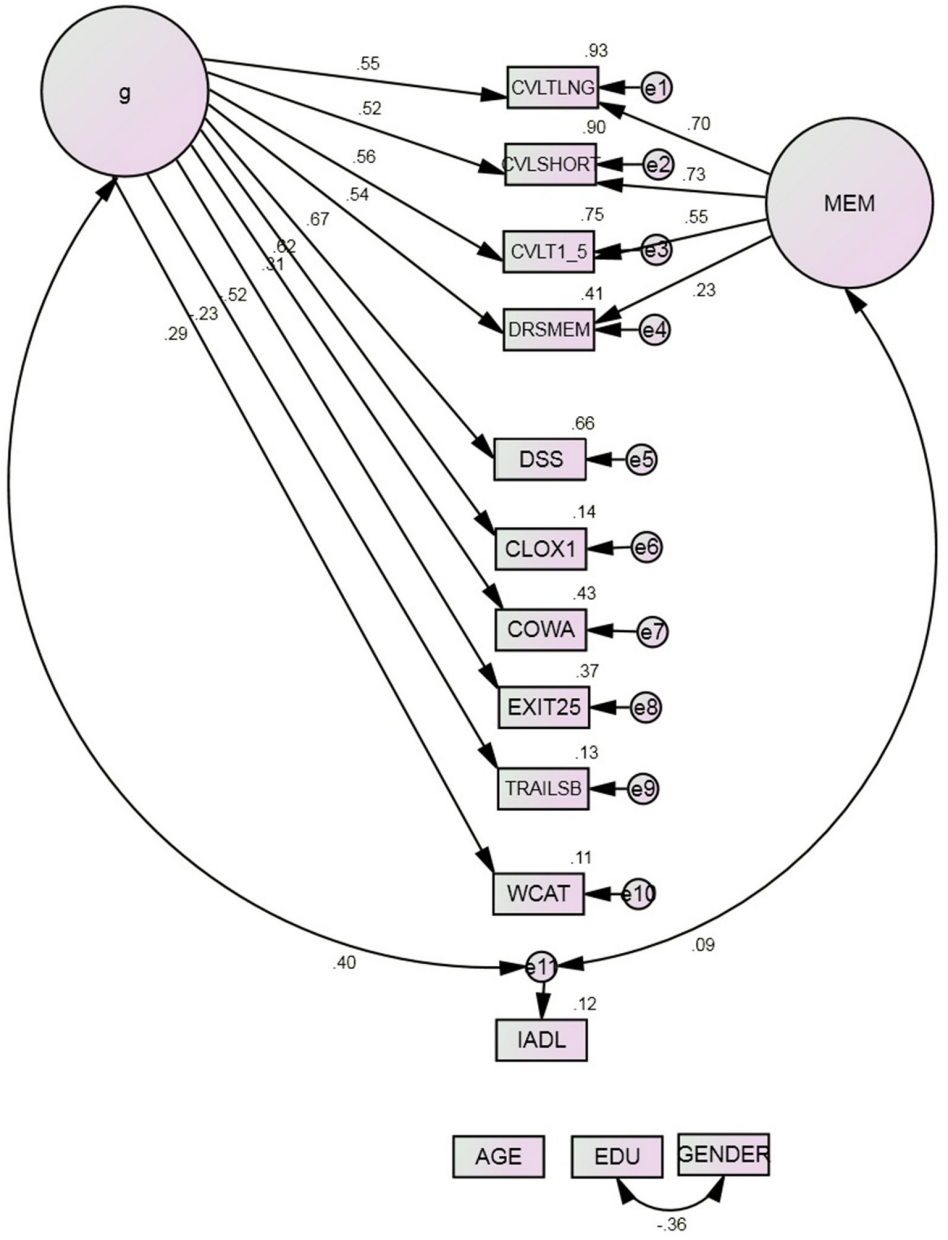
Model 3b^*^. COWA, Controlled Oral Word Association Test; CLOX1, clock drawing to command; CVLT, California Verbal Learning Test; 1–5, Summed learning trials 1–5, SHORT, immediate recall, LNG, delayed recall; DRS: MEM, Mattis Dementia Rating Scale Memory subscale; DSS, WAIS-R Digit Symbol Substitution; EDU, Education; EXIT25, Executive Interview; IADL, Instrumental Activities of Daily Living; TrailsB, Trail-making Test Part B; WCAT, Wisconsin Card Sorting Test categories achieved. ^*^All observed indicators are adjusted for age, education, and gender (paths not shown).

In the adopted Model 3b (Figure 3), g was indicated significantly by all the cognitive measures (all *p* ≤ 0.002) ranging from Trails B (*r* = −0.23, *p* = 0.002) to DSS (*r* = 0.66, *p* < 0.001). MEM's factor loadings were slightly attenuated by g's creation, ranging from *r* = 0.23 (DRS:MEM) to *r* = 0.70 (CVLT:Long). g was significantly correlated with IADL (*r* = 0.40, *p* < 0.001). MEM had no significant association with that variable (*r* = 0.09, *p* = 0.261). Thus, g both mediates MEM's unadjusted association with IADL and better fits the variance in our putative ECF measures than would an EF domain-specific factor, whether adjusted for g or not.

Model 4 parses Spearman's g into two fractions (Figure 3). d is indicated by IADL and the cognitive performance measures. g′ (i.e., d's residual in Spearman's g) and MEM are indicated only by cognitive performance measures. This arrangement had excellent fit, and fit the data significantly better than any previous model (Table 3). d was significantly indicated by all the cognitive measures except WCAT (*r* = 0.10, *p* = 0.30) and Trails B (*r* = 0.05, *p* = 0.63). WCAT and Trails B loaded significantly on g′ (both *p* ≤ 0.002) as did all the other cognitive measures, ranging from CLOX1 (*r* = −0.27) to COWA (*r* = −0.62, both *p* < 0.001).

However, by definition, g′ had no association with IADL. In contrast, d was associated strongly with IADL (*r* = 0.65, *p* < 0.001). Independently of their associations with d, no cognitive performance measure was significantly associated with IADL, i.e., through their residuals. Thus, WCAT and Trails B had no significant associations with IADL at all.

**Figure 4:**
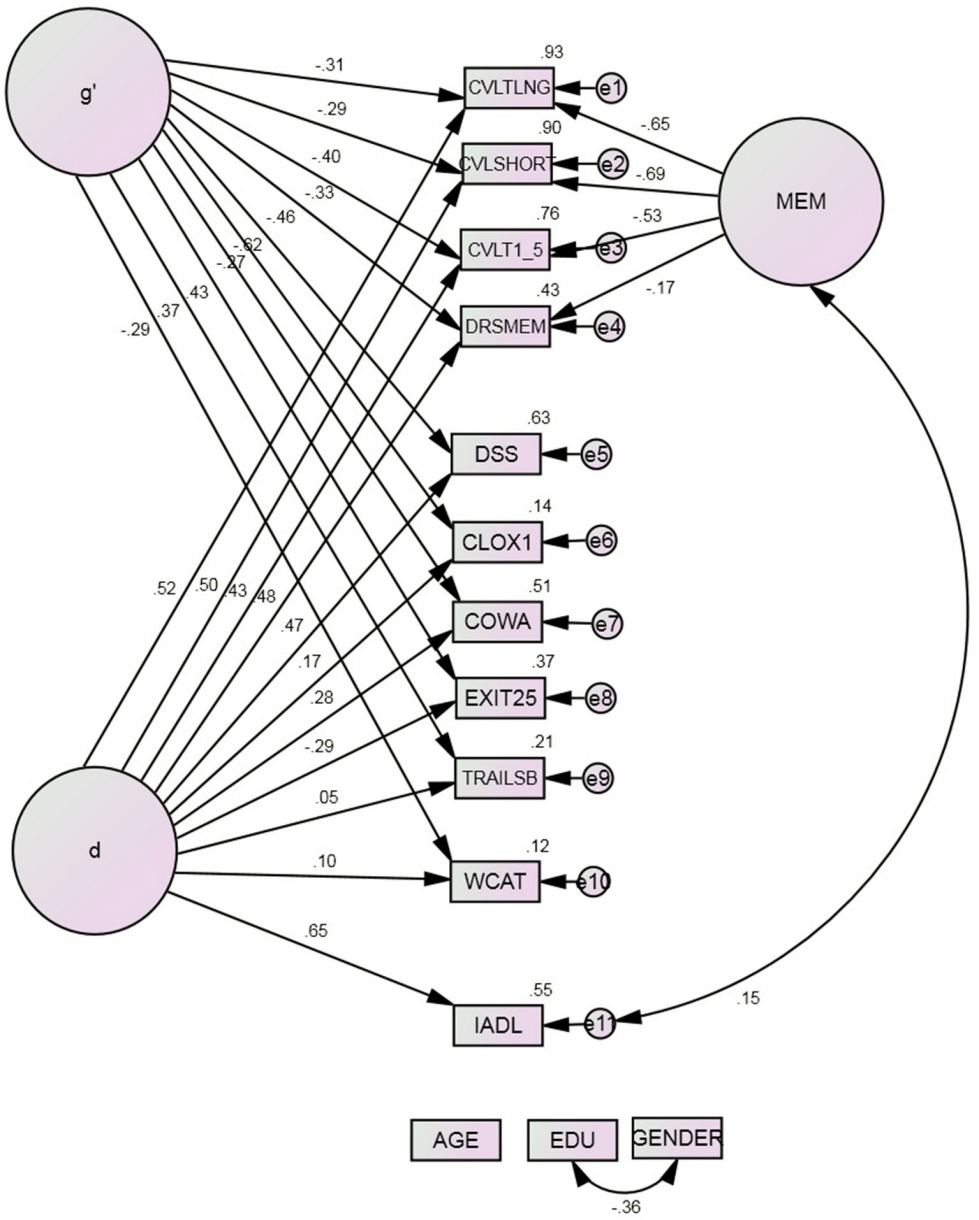
Model 4^*^. COWA, Controlled Oral Word Association Test; CLOX1, clock drawing to command; CVLT, California Verbal Learning Test; 1–5, Summed learning trials 1–5, SHORT, immediate recall, LNG, delayed recall; DRS: MEM, Mattis Dementia Rating Scale Memory subscale; DSS, WAIS-R Digit Symbol Substitution; EDU, Education; EXIT25, Executive Interview; IADL, Instrumental Activities of Daily Living; TrailsB, Trail-making Test Part B; WCAT, Wisconsin Card Sorting Test categories achieved. ^*^All observed indicators are adjusted for age, education, and gender (paths not shown).

There were no significant intercorrelations amongst the residuals of the final three latent constructs' indicators, in Model 4. Specifically, none of the ECF measures' residuals were significantly correlated. This finding closes the door to the possibility of one or more unmodeled factors, including EF or processing speed. Since the modeled factors explain a minority of the variance in most ECF measures (Figure 4), their uncorrelated residuals may reflect measure specific “measurement error.” By definition, the three latent variables d, g′, and MEM were orthogonal to each other and could not be intercorrelated.

## Discussion

In this analysis, we have confirmed the relatively strong association between the EXIT25, and IADL, in a multivariate regression model. The EXIT25 contributed significantly to IADL independent of memory measures and a battery of other EF measures. This is consistent with several previous studies in a wide range of samples (Chan et al., 2006; Lewis and Miller, 2007; Pereira et al., 2008), including this one (Royall et al., 1998a, 2000, 2004, 2005a,b).

Together, cognitive measures and covariates explained a respectable fraction of IADL variance. However, the model did not fit the data well. The SEM approach forces our attention to the quality of a model's fit, not merely the significance of its parameters and the total variance explained in its dependent variable. In every case, the introduction of latent variables fit the data better than did our initial multivariate regression approach.

Model 2 has confirmed our apriori assumptions about the domain specific face validity of our cognitive battery. All the memory measures loaded significantly on the latent construct “MEM.” All the executive measures loaded significantly on the latent construct “EF.” These factors were not significantly associated with each other. As we expected, EF was more strongly associated with IADL than MEM, which was weakly associated with that construct.

However, subsequent models with better fit have forced us to abandon the EF construct. The introduction of g′ and d provide a much better fit, and the absence of significant intercorrelations among their indicators' residuals closes the door to the possibility of unmodeled alternative factors (e.g., processing speed, etc.).

Models 3b and 4 suggest that EF measures have no association with IADL independent of general intelligence and specifically its subfraction δ. Model 4 demonstrates that EF measures have no special or unique association with IADL, even through d. d is also indicated by memory tasks, and they load more strongly on d than any executive measure.

Independent of d, EF measures cannot be associated with IADL, either individually (through their residuals), or via g′ (by definition). WCAT and Trails B load only on g′ and thus have no association with IADL at all. This is consistent with their failure to contribute significantly to IADL independently of the EXIT25 and other EF measures in Model 1.

Both findings also replicate our earlier factor analysis in this dataset (Royall et al., 2003). In that analysis, the variance in a battery of EF measures was empirically distributed across three factors. The first (28% of variance) was indicated by CLOX, COWA, DSS, and the EXIT25. The second (24.2% of variance) was uniquely indicated by the WCST and its subtasks. The third (12.4% of variance) was indicated uniquely by Trails B. Only the first factor was associated with IADL. The fact that d and g explain so little of the variance in our battery of otherwise non-correlated measures suggests that each EF measure may have considerable “measurement error” associated with it.

Duncan and others have previously associated g with frontal structure and function (Duncan and Owen, 2000; Duncan et al., 2000; Choi et al., 2008; Gläscher et al., 2010). Similarly, several of our EF measures have been associated with frontal structure and /or function (Royall et al., 2007; Royall, 2011). However, Model 4 demonstrates that the variance in our EF indicators is distributed across two orthogonal latent factors, d and g′. Neither is specifically associated with EF, as both are significantly indicated by also by memory tests. It is an empirical question which, if either latent construct can mediate g's observed association with frontal structure and /or function. Our dataset cannot address that question.

Because g (Model 3), g′ and d (Model 4) have been adjusted for memory-specific task performance (i.e., MEM), it could be argued that the loadings of memory tasks on the first three latent constructs reflects the “executive” fraction of those measures' variance (e.g., “Working Memory”). Working Memory has been related to “updating” and can be associated with measures of intelligence (Friedman et al., 2008).

However, only d is associated with IADL. Working Memory has previously been associated with IADL (Lewis and Miller, 2007) and d is more strongly indicated by memory tasks than by executive ones. Moreover, d and g′ are orthogonal to each other. Thus, they cannot both be “executive,” and if g′ were to be identified as the true executive factor (after all, it is most strongly loaded by COWA and the only factor associated with Trails B and WCAT) then EF can again have no impact on IADL.

d uniquely accounts for a sizable fraction of IADL's variance, and explains more variance in IADL than did the ECF factor in Model 2, or indeed the entire battery in Model 1. d is a homolog of δ, our latent dementia proxy. δ and its homologs are strongly and specifically associated with clinical dementia status, as measured by the Clinical Dementia Rating Scale (Hughes et al., 1982; Royall et al., 2012a,b; Royall and Palmer, 2013, 2014). Even in this non-demented cohort, the interindividual variance in δ scores predicts longitudinal change in ECF measures, specifically the EXIT25 and Trails B (but neither WCAT nor the CVLT) (Royall and Palmer, 2012). The fact that δ predicts longitudinal change in Trails B in this very cohort suggests that Trails B's failure to load on d in this analysis may be an artifact of its baseline distribution, which is skewed and subject to floor effects. In longitudinal analyses, each subject is its own control.

In contrast to Spearman's g, δ has been associated with atrophy in the Default Mode Network (DMN) (Royall et al., 2012b, 2013). The DMN is associated with a subregion of the frontal lobe (i.e., a small portion of the dorsolateral prefrontal cortex), but also with subregions of the temporal lobe, the parietal lobe, the cingulate gyrus and the hippocampus (Buckner et al., 2008). The latter may explain the relatively strong loadings of memory measures on d. Thus, it seems unlikely that d would localize to the frontal cortex, as might be expected of an “executive” construct (although specific frontal localizations have in fact not been shown for many executive measures).

In short, the associations between the EF factor, or its indicators and IADL are mediated uniquely through d, i.e., a fraction of Spearman's g. This result is similar to an analysis by Salthouse et al. (1996) of age's influence on cognitive task performance. They found moderately strong age-related declines on a battery of tests that included the WCST, Trails-B, and DSS, among others. However, correlation-based analyses revealed that the age-related effects on different measures were not independent. Instead, the effect of age was observed specifically in the fraction of variance (averaging 58%) shared across all the observed measures (i.e., “g”). Thus, g′ and δ may also mediate age-specific effects on ECF measures. This would explain age's broad effects on cognitive performance, relatively strong effects on “ECF” measures, and the disabling character of those effects (if mediated through δ).

On the other hand, aging is also characterized by a “de-differentiation” of cognitive test performance (McArdle et al., 2002). This may favor the demonstration of global constructs such as g, g′, and δ. It remains to be seen if a δ homolog would mediate the association(s) between one or more EF factors and IADL in healthy younger adults. One potential obstacle to such a study would be selection of a valid IADL measure. The informant-rated IADL measure we used here may have floor effects in highly functioning populations. Nevertheless, δ is not very sensitive to its IADL target, and has similar psychometric properties regardless of the target IADL indicators used to date (Royall and Palmer, 2012, 2013, 2014; Royall et al., 2012a,b; Gavett et al., 2014).

Our dataset is further limited by other issues. It does not contain measures of supposedly fundamental executive tasks (i.e., inhibition, categorization, and updating) (Miyake et al., 2000). Such measures are arguably less prone to measurement error than the “complex” ECF measures we have employed. They, and other executive tasks (e.g., set-shifting and delayed matching to sample) have been associated with frontal lobe lesions and structures. However, such low level cognitive abilities (which can be demonstrated in chimpanzees for example, at an estimated three year old human intelligence equivalent) (Moriguchi et al., 2011) may not be representative of the emergent ECF that characterizes adult human action.

It is arguable that δ cannot be demonstrated in any animal that is incapable of IADL (by definition). This may have a biological explanation. The human brain, uniquely among primates, exhibits frontal networks that extend beyond the frontal lobe (including the DMN). The frontal networks of other primates are localized to that structure (Wey et al., 2013). Frontal tasks not-related to IADL, and /or demonstrable in animals incapable of IADL, are arguably not “executive” but merely “frontal.” They may be associated with δ in humans, but then so might any cognitive performance measure, whether executive or non-executive, and whether localizable to the frontal lobe or not. Regardless, their demonstration and functional localization to frontal structures in animals incapable of IADL will not be associated with δ, by definition.

Friedman et al. (2008) have demonstrated the existence of a latent “Common” EF factor, that is indicated by all basic EF measures, as are g, g′, and δ /d. Friedman et al. distinguished their Common factor from both intelligence and processing speed. However, they did not try to associate their Common factor with non-executive indicators, and so its specificity to EF is undemonstrated, as is its association with IADL, and therefore δ.

Ironically, the Common factor's independence from intelligence suggests that it may indeed be more likely to correspond to d in this analysis than to g′, as g′ would be expected to correlate more strongly with observed performance on intelligence measures. Friedman et al. also observed that a theorized “Inhibition” factor collapsed after the Common factor's introduction. That is consistent with EF's collapse in our analysis after the introduction of g.

Second, our battery is limited in its ability to assess “processing speed.” Trails B is our only timed test, although some authors associate performance on the DSS with this construct. This limits our ability to speak to processing speed as a determinant of IADL. However, such a factor is unlikely to attenuate δ's association with IADL because processing speed is an intermediate “domain-specific” factor (like MEM and EF in this analysis) and thus taps a compartment of variance in cognitive performance that is orthogonal to g (and therefore both g′ and δ). Had our battery been better designed to assess processing speed, we expect it would have robbed MEM of its relatively weak association with IADL rather than d.

Finally, this analysis is limited to cross-sectional data. At baseline, the FHS cohort was cognitively normal for its age, relatively highly functioning and non-institutionalized. Few subjects can be expected to have been clinically demented, although a sizeable fraction might have had “mild” neurocognitive disorders. Thus, restricted range and floor effects on some measures may have affected our analysis.

Clinical dementia status was never formally adjudicated in this cohort. Never the less, we have demonstrated that there is significant variability with regard to the cohort's longitudinal rates of change in cognitive performance over time (Royall et al., 2005a). These changes are clearly related to concurrent declines in functional status (Royall et al., 2004) suggesting aging-related declines in δ-specific variance. In fact, we have shown those associations to be mediated through δ (Royall and Palmer, 2012). Gavett et al. (2014) report that the six-year prospective longitudinal change in δ scores (Δδ) correlates strongly (*r* = −0.94, *p* < 0.001) with change in dementia severity, as rated by the Clinical Dementia Rating scale (CDR) (Hughes et al., 1982). Similarly, in the Texas Alzheimer's Research and Care Consortium (TARCC), δ's intercept and slope explain 79% of the variance in four year prospective dementia severity, independently of baseline dementia severity, g′ and Δg′ [Palmer and Royall (ICAAD abstract), 2013]. If ECF (as distinct from EF) is synonymous with δ then it likely is the major cognitive determinant of dementia status in humans and dementia, in turn, may be limited to structural and functional pathologies of the DMN (Royall et al., 2002b, 2012b).

In summary, we have used a latent variable approach in an SEM framework to construct a well fitting model that suggests that the variance in a battery of well validated “executive” measures cannot be related to a domain specific “executive” factor independent of Spearman's general intelligence factor, g. Moreover, no cognitive performance measure in our battery can be associated with IADL independently of a certain fraction of that latent construct, i.e., d. d, as a δ homolog, is likely to be associated specifically with the structure and function of the DMN. That network extends well beyond the frontal lobe, and can be related only to certain subregions in that structure. This underscores the importance of disentangling “EF” from “frontal function” (Royall et al., 2002a).

Although we again confirm that memory specific variance has no association with IADL (and by extension with dementia), memory performance measures do contribute significantly to g (as should all cognitive performance measures) and its subparts: g′ and d. Only their contributions to d would be salient to functional outcomes and dementia. However, memory tasks are more strongly associated with that construct than were most “ECF” measures. It is the distribution of memory task performance across three latent constructs, two of which are irrelevant to IADL and dementia case finding that weakens their performance as predictors of IADL. In contrast, a larger share, if not the majority of variance in most putative “ECF” measures (but neither Trails B nor WCAT), is invested in δ. This explains the relatively strong associations between putative “ECF” measures, IADL and dementia status in past studies. Regardless, δ homologs should have even greater potential for dementia case-finding, although they are neither indicated solely by ECF measures, nor likely to localize specifically to the frontal lobe.

### Conflict of interest statement

The authors declare that the research was conducted in the absence of any commercial or financial relationships that could be construed as a potential conflict of interest.
